# Architecture of a Service-Enabled Sensing Platform for the Environment

**DOI:** 10.3390/s150204470

**Published:** 2015-02-13

**Authors:** Alexander Kotsev, Francesco Pantisano, Sven Schade, Simon Jirka

**Affiliations:** 1 Scientific/Technical Project Officer at European Commission-Joint Research Centre, Institute for Environment and Sustainability, Digital Earth and Reference Data Unit. Via E. Fermi, Ispra 2749 I-21027, Italy; E-Mails: francesco.pantisano@jrc.ec.europa.eu (F.P.); sven.schade@jrc.ec.europa.eu (S.S.); 2 52°North Initiative for Geospatial Open Source Software GmbH, Martin-Luther-King-Weg 24 48155 Münster, Germany; E-Mail: jirka@52north.org

**Keywords:** environmental sensing, interoperability of sensor devices, Internet of Things, INSPIRE, RaspberryPI, OGC, Sensor Observation Service

## Abstract

Recent technological advancements have led to the production of arrays of miniaturized sensors, often embedded in existing multitasking devices (e.g., smartphones, tablets) and using a wide range of radio standards (e.g., Bluetooth, Wi-Fi, 4G cellular networks). Altogether, these technological evolutions coupled with the diffusion of ubiquitous Internet connectivity provide the base-line technology for the Internet of Things (IoT). The rapid increase of IoT devices is enabling the definition of new paradigms of data collection and introduces the concept of mobile crowd-sensing. In this respect, new sensing methodologies promise to extend the current understanding of the environment and social behaviors by leveraging citizen-contributed data for a wide range of applications. Environmental sensing can however only be successful if all the heterogeneous technologies and infrastructures work smoothly together. As a result, the interconnection and orchestration of devices is one of the central issues of the IoT paradigm. With this in mind, we propose an approach for improving the accessibility of observation data, based on interoperable standards and on-device web services.

## 1. Introduction

A rapidly increasing number of interconnected sensing devices, which are organized within sensor networks of different structure and complexity, provide new knowledge about our environment. The generation of information by citizens through smartphones and all sorts of different gadgets is fundamentally changing the traditional ways of data collection. At the same time we witness record numbers of hardware manufacturers, as well as a wide array of platforms, communication protocols and technologies. In fact, the number of devices connected to the Internet has already exceeded the size of the world population in 2008, and is expected to reach 50 billion by 2020 [1]. Such a technological evolution has laid the foundations for the Internet of Things (IoT), that has been defined by a European Commission report as “a world-wide network of interconnected objects uniquely addressable, based on standard communication protocols” [2]. The IoT and the proliferation of mobile sensors provide the building blocks that are required to build monitoring platforms. As a typical example of pervasive technology, monitoring platforms have initiated a new generation of wireless sensor networks with wide range of purposes [3]. Amongst main applications, the collective use of sensing devices—organized within the IoT, and their virtual representation through the web of things—lead to a mosaic that improves the monitoring and understanding of our complex environment. This creates a revolution in all environmental sciences, similar to the one generated by the use of satellite remote sensing in the 1970s [4]. Furthermore, the IoT and the rapid shift of the software industry towards the concept of service-oriented architectures (SOA) is blurring the boundary between the physical and virtual realities, thus providing a fertile ground for a new breed of applications which are aware of the real world [5].

The above vision of future environmental sensing can only be successful if a larger than ever number of heterogeneous devices and infrastructures work smoothly together. Hence, it becomes essential to interconnect devices into interoperable systems [6]. However, particularly in respect to environmental monitoring infrastructures, the interconnection and orchestration of devices are still constrained by at least the following interdependent factors:

- Difficult discovery of environmental sensor devices and networks, due to the lack of metadata and services which expose them [7];
- Spatial/temporal mismatch of observations and measurements—deriving data from unevenly distributed monitoring stations that do not always form networks is causing difficulties in data reuse for initially unintended purposes [8];
- Lack of interoperability between components (e.g., measurement devices, protocols for data collection and services) of acquisition and dissemination systems [9,10];
- Information silos, created by the use of standalone vocabularies that are bound to particular environmental domains, such as hydrology and air quality [11];
- Proprietary solutions for logging sensor measurements, which require custom code to be wrapped around the manufacturer's software development kit [12].

Given the above context, this paper proposes an approach to enable the interoperability of environmental sensors through the integration of on-board storage and web service capabilities. Through the proposed approach observation data can easily be combined and reused, thus benefitting users in the following direction:

- Out-of-the box (plug-and-play) deployment of sensor nodes;
- Independence from particular platform implementations;
- Simplified provision of sensor data access;
- Reduced efforts for data integration, and thus a lower barrier for users to rely on the offered environmental data.

Following the introduction of the key concepts, the second section of the manuscript provides overview of the factors and preconditions that underlie the proposed approach. For that purpose we look at sensors from an IoT perspective and present our views on the enabling technology. The third section describes our proposal for an interoperable platform for environmental sensing, which includes a general overview together with several well-established standards and interoperable technologies which, when used together act as enablers of the IoT. Then we provide our results from an implementation of an according platform as a proof of concept. In the fourth section we provide our perspective on the added value of the proposed architecture, combining sensing and data serving through the same device, thus enabling the use of web services directly from a sensor node. We conclude with a discussion on the existing challenges towards fully-interoperable sensor networks and our future research direction.

## 2. Enabling Factors for Interoperable Sensing of Our Environment

The design of an environmental sensing platform with interoperability as a central feature faces two main challenges:

- The unified management of different data and metadata formats; and
- The deployment of heterogeneous sensors and the integration of their interfaces.

As a matter of fact, sharing environmental sensing information across heterogeneous networks demands a solid common model to represent the exchanged messages based on commonly adopted rules. In order to implement those, recent scientific efforts have been poured on the definition of interoperability protocols, on several levels of abstraction.

Significant advances were reached in standardizing wireless physical interfaces [13] so as to effectively transfer the information based on a set of syntactic and semantic rules on the respective metadata. These works distinguish a standardized Physical (PHY) and Medium Access Layer (MAC) related with low-rate personal area networks (e.g., IEEE Std. 802.15.4, 2011) [14].

Sensor network interoperability has been promoted by several initiatives in the recent past, including those within the Open Geospatial Consortium (OGC) and the International Telecommunication Union (ITU). In essence, the OGC standard compliant networks (i) facilitate the design of service oriented architectures (SOA); (ii) enable the reuse of available information through spatial data infrastructures; and (iii) provide a unified framework for sensor measurements [15]. In higher layers, (e.g., the application layer), sensor network standards such as the OGC standards provide essential conceptual structures. They provide the means to describe and exchange information through reusable syntactic metadata, and by including standard languages and meta-languages (e.g., XML). An overview of the stack of standards provided by the OGC for sensor networks is provided in Section 2.4.3. Similarly, the ITU has issued a series of recommendation for the design and development of IoT architectures, by identifying the high-level requirements and providing a reference model for interoperability and network efficiency [16,17].

Clearly, the development of environmental sensing platforms, which are interoperable at the application layer, demands a top-down approach by design. This has to be carefully considered-right from the initial design and planning. Despite the increased complexity, application-layer-interoperability is desirable because it ultimately enables the exchange of structured information across different platforms, in a simpler, secure and reliable manner. In this section, we review the interrelated factors which allow the creation and deployment of such interoperable sensing devices in large numbers.

### 2.1. Wireless Networking Availability and Interoperability

The evolution of wireless networks has been driven by the ever-increasing demand for pervasive connectivity, as demonstrated by the expected ten thousand-fold increase in mobile traffic data and a hundred-fold increase in the number of devices with networking capabilities [18,19]. To address such a data deluge, future network infrastructures will witness the combination of existing and emerging systems, such as cellular communication networks (e.g., Long Term Evolution (LTE)) and Wi-Fi, coupled with novel technologies designed to meet the quality of service requirements.

In fact, on the one hand, Wi-Fi will retain its main role for low-cost, best effort data delivery, notably in indoor scenarios. On the other hand, the upcoming generation of mobile cellular networks will cater for the high-traffic, low-latency, low-energy requirements, by mainly focusing on outdoor urban scenarios.

In addition to the urban context, several ambitious projects, such as the Google Loon project [20] and the drone-based Internet programme of Facebook [21], are investigating over-the-air network infrastructures for providing large bandwidth Internet coverage to remote regions or areas with little or no information and communication technology (ICT) infrastructure. Clearly, such projects catalyse the introduction of new IoT devices and monitoring applications.

In such a complex scenario, interoperability and network efficiency functionalities have to be considered at all layers of the communication protocol stack in a cross-hierarchical approach [22]. As a matter of fact, the legacy Internet network was primarily designed for human interaction and communication. Here, as the humans were responsible for the semantic information processing, the required interoperability was mainly limited to the communication protocols, such as the unanimously adopted Internet Protocol. However, in a (near) future network scenario in which machine-to-machine communication generates most of the traffic, semantic and cross-layer interoperability become crucial.

### 2.2. Accessible and Low-Cost Mobile Computing

Mobile computing is becoming increasingly available, while the prices of hardware are constantly decreasing. Projects such as RaspberryPI, Arduino, Fritzing, Wiring and Waspmote provide very cheap modular hardware components, which can easily be programmed and configured through standard free and open-source software (FOSS). As the applications of sensor networks are diverse, the hardware created for the purposes of the IoT varies significantly. For instance, some hazard monitoring networks are designed to operate autonomously for long periods of time, while decentralized computing networks aim at top performance at the cost of best-effort battery life. In this section, we provide a short overview of the hardware components which we see as most promising for the purpose of establishing an interoperable environmental sensing platform.

#### 2.2.1. RaspberryPI

RaspberryPI [23] is a low energy consumption credit-card-sized computer which costs as little as 25 Euro. The device was initially created for teaching programming and computing in schools. A detailed overview of the RaspberryPI is provided by [24]. The device is bundled with a fully featured Linux distribution in contrary to the traditional notion that operating systems for wireless networks are very different from those for desktop/laptop computers [25].

RaspberryPI is extensively used for purposes going far beyond the initial educational application. The reasons are a combination of low energy consumption and high computational power and affordability. The actual applications are very diverse and range from controlling robots, environment monitoring drones, high altitude atmosphere observation and gaming platforms.

#### 2.2.2. Arduino

Arduino [26] is an open electronics prototyping platform with components which can extend its functionality to monitor the environment for parameters such as air quality, noise, illumination, temperature. The prototyping platform is modular and designed with the overall idea to provide cost efficient components designed to become part of the IoT. The Arduino hardware is considerably less computationally capable than RaspberryPI. Arduino boards can be connected to a RaspberryPI through a shields connection bridge. Through that one can use any of the shields, boards and modules designed for Arduino in Raspberry PI, thus making the most of both products.

#### 2.2.3. Waspmote

Waspmote [27] is a platform designed explicitly with the IoT in mind. It focuses on the creation of sensor nodes with low energy consumption which are able to work independently for long periods of time (within the range of one to five years). Waspmote reuses the same development environment as Arduino, so code developed for an Arduino node can also be used in this device making minor or no adjustments. There are at present over 60 different sensors which can be connected to the Waspmote.

### 2.3. Free and Open-Source Software (FOSS)

In this study, we reviewed and tested numerous products which might potentially enable interoperable environmental sensing. We exclusively focused on open source solutions, to take advantage of the numerous opportunities for sharing, sharing, modification, reuse, and redistribution of the software products [28]. Furthermore, we consider that this would allow reuse and easy integration between the different components needed for a sensing platform (operating system, storage, service, *etc.*). Table 1 provides an overview of the studied products together with our evaluation of their advantages and disadvantages.

### 2.4. Interoperable Service Oriented Architecture

SOA is defined as an environment where loosely-coupled network resources are made available as independent services, which can be accessed without preliminary knowledge of their underlying implementation platform and exist autonomously yet not isolated from each other [29]. Clearly, interoperability is of critical importance for services to be able to be combined. That is why unifying the ways environmental sensing devices communicate and encode data is tightly linked to the extensive use of commonly recognized standards. Initiatives of international standardization organizations such as International Standardization Organization (ISO), Institute of Electrical and Electronics Engineers (IEEE), International Telecommunication Union Standardization Sector (ITU-T), Open Geospatial Consortium (OGC), and many others target a broad spectrum of topics on different conceptual levels that together provide a backbone for sensor network interoperability.

#### 2.4.1. Internet of Things Standards

##### Networking Standards

When considering cross-system interoperability, it must be noted that Wi-Fi exhibits many appealing features, due to its openness, vendor-neutrality and pervasive availability in off-the-shelf electronic devices. Nevertheless, Wi-Fi is yet unsuitable for sensor communication, especially in dense networks where low power, security, and reliability are key requirements [30]. As a result, in order to achieve these properties, there is a need for redesigning and extending the Wi-Fi functionalities, as recently demonstrated by the quickly emerging communication protocols proposed for wireless sensor networks (WSN) PHY and MAC layers. In this respect, three de facto standards, which are described below, are currently emerging: Thread, Z-Wave and Bluetooth Smart (also known as Bluetooth Low Energy-BLE) [31].

The communication protocol proposed by the Thread group [32] is based on 6LoWPAN, a low-power wireless protocol that delivers IPv6 over the IEEE 802.15.4 radio, which is also used for ZigBee sensors nodes. Thread extends the functions of 6LoWPAN specifically for mesh network topologies, as those commonly considered in light of Internet of Things use cases. The extended functions include coverage optimization, battery life optimization and end-to-end communication security. In addition to bringing mesh functionalities to 6LoWPAN, Thread adds a layer of security, enables point-to-point communications, and provides schemes for optimizing battery life. Network architectures have been specifically developed to work with IPv6, while devices will need a chip with a standard 802.15.4 radio to use Thread.

Bluetooth Smart and Smart Ready [33] represent the evolution of the commercialized Bluetooth version towards dense sensor networks with stringent energy efficiency requirements. Bluetooth Smart radio exclusively operates on a single mode, which is a configuration that optimizes the battery life and consumes between 1/2 and 1/100 of the conventional Bluetooth version. However, since Bluetooth Smart is not backwards compatible with other Bluetooth devices, the Bluetooth Smart Ready standard has been proposed as it allows dual mode transmissions, *i.e.*, both traditional Bluetooth wireless connections and the low-energy connection mode. Both Bluetooth Smart and Smart Ready versions operates on the 2.4 GHz spectrum, and exhibit a high degree of interference immunity, by leveraging adaptive frequency hopping capability.

Z-Wave [34] is a communication protocol home network, proposed by the eponymous Z-Wave Alliance [35], a consortium of leading manufacturers of control appliances. The Z-Wave protocol is designed for security and efficient energy management. Unlike Thread and Bluetooth, Z-Wave operates in the sub-GHz spectrum (*i.e.*, 900 MHz), thus it is able to avoid interference in the 2.4 GHz band. Nevertheless, the Sub-1GHz transmissions require a larger antenna set, usually 2.5 times larger, which limits the range advantage of the Sub-1GHz technology.

As the above communication protocols are pushed by several consortia of ICT manufacturers, each one addressing a specific market sector, the success of each solution is linked to the suitability of the network protocols to the specific applications. As a matter of fact, the criteria for the choice of a suitable network communication protocol include considerations on network reliability, security, fault recovery capabilities, chipset cost, and required human interaction, amongst others.

##### ITU Standardization Initiative

The International Telecommunication Union (ITU) is a United Nations agency, specialized in information and communication technologies. The main mission of the agency is the allocation of radio spectrum and satellite orbits, as well as the development of technical standards which ensure that networks and technologies seamlessly interconnect. The standardization activities carried out by ITU Standardization sector (ITU-T) have a fundamental role in order to ensure a secure and autonomous deployment of IoT. The standardization activities carried out by ITU Standardization sector (ITU-T) have a fundamental role in order to ensure a secure and autonomous deployment of IoT, while more pragmatic aspects pertaining guidelines and recommendations for architecture design are covered within the Joint Coordination Activity on Internet of Things (JCA-IoT) [17].

##### IOT-Architecture

IoT-A is a pan-European project dedicated to the establishment of a generic architecture for the Internet of Things, departing from existing architectures and solutions, and identifying reusable components, which are further stacked into solutions in accordance with the IoT-A vision, and a generic Reference model and Reference architecture. The IoT-A vision promotes the need for a high level of interoperability to be achieved at communication, service and information level, going across various platforms, but being established on a common IoT-A grounding [8].

##### OpenIoT Platform

The European OpenIoT research project [36] has established an open source middleware for getting information from sensor nodes in a vendor-independent manner, at the same time seeing the IoT an extension to cloud computing [37]. OpenIoT is addressing a wide spectrum of interrelated scientific and technological issues, such as: (i) Middleware for sensor networks; (ii) Ontologies, semantic models and annotations for representing IoT components (iii) Cloud computing [38]. Through this the OpenIoT project and the associated software platform addresses the rising need for data fusion and data integration functionalities, with the aim of creating new value-added services for the IoT case. Such functionalities will be enabled by two main conditions: (i) enhancing data accessibility and (ii) by fostering multi-standard interoperability. The first of the above conditions mainly addresses the issue of data openness-according to which, the IoT services grant third parties access to IoT sensors data, provided that such information has been processed according to a security and privacy policy. For example, in such a setting, additional efficiency gains become feasible as multi-source information can be collectively exploited for a common optimization goal.

With respect to the second condition on interoperability, one should note that IoT is a multi-vendor, multi-standard ecosystem, which is designed for device connectivity, and not necessarily for compatibility. In such a scenario, initiatives promoting the use of open source software or open-linked data techniques (e.g., SPARQL Protocol and RDF Query Language) are a promising trend as they would allow overcoming the inherent limitations posed by standards or proprietary solutions. That is why projects such as OpenIoT represents a joint effort of prominent open source contributors towards enabling a new range of open large scale intelligent IoT applications according to a cloud computing delivery model.

#### 2.4.2. Spatial Data Infrastructures

From a spatial data infrastructure (SDI) perspective sensor information is seen as the next step for building a mosaic of information that will better allow us to understand and manage the environment. The handling of sensor data however presents a new challenge for SDIs as they traditionally emerged from the geospatial information systems (GIS) domain, and thus focus on the locational characteristics of data. Despite the fact that the number of devices connected to the Internet is rapidly increasing in terms of data [1], data handling is not challenged by location of these devices, but by the observations and measurements that they create. Considering the integration of temporal data within an SDI, Europe achieved several significant advancements as part of the world's largest data harmonization effort for an environmental information infrastructure-the Infrastructure for Spatial Information in Europe (INSPIRE). INSPIRE is backed up by a set of legal acts (Directive 2007/2/EC of the European Parliament) [39] and rolls out detailed plans for establishing a Europe-wide data infrastructure for environmental and environment-related data. Relevant work on sensors covers both, data encoding [40] and network services [41], together providing all necessary means for “plugging” spatio-temporal data into SDIs, thus enabling its use and reuse combined with other relevant resources.

#### 2.4.3. Sensor Web Enablement Standards of OGC

The OGC is an international standards organization with more than 450 members including research institutions, governmental organizations, non-governmental bodies and commercial organizations. They collaborate for consensus-based creation and consequent implementation of standards in the geospatial data and services domain, including the management and integration of sensor networks [42].

A particular bundle of standards of the OGC known as Sensor Web Enablement (SWE) framework refers to web accessible sensor networks and archived sensor data that can be discovered, accessed and—where applicable—controlled using open standard protocols and interfaces [43]. A central goal of this standards framework is the integration of sensors and their observations into SDIs.

**Figure 1.**
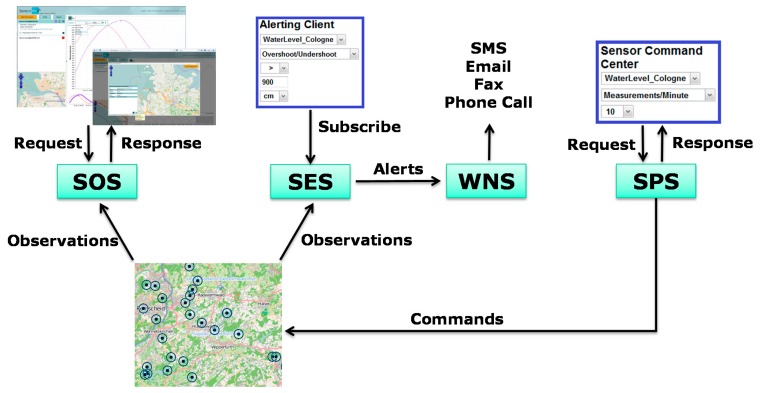
Stack of standards part of the OGC Sensor Web Enablement.

Figure 1 provides overview of the components which together form the SWE suite. Those include the Sensor Observation Service (SOS), the Observations and Measurements standard (O&M), the Sensor Model Language (SensorML) specification (sensor metadata), the Sensor Event Service (SES), and the Sensor Planning Service (SPS), formerly Sensor Alert Service (SAS). The Sensor Observation Service (SOS) is the component of SWE which is intended for the provision of observation and measurement data with spatial and temporal reference relying on the O&M data model and encoding. The SOS interface is well suited for flexible delivery of data with significant spatio-temporal variability. The rest of the SWE standards cover different aspects relevant for the establishment and use of sensor networks ranging from configuring and controlling sensors/sensor networks to triggering alerts and notifications in an interoperable manner. Further details on SWE are provided in [43,44].

## 3. Architecture for Interoperable Environmental Sensing

### 3.1. General Considerations

**Figure 2.**
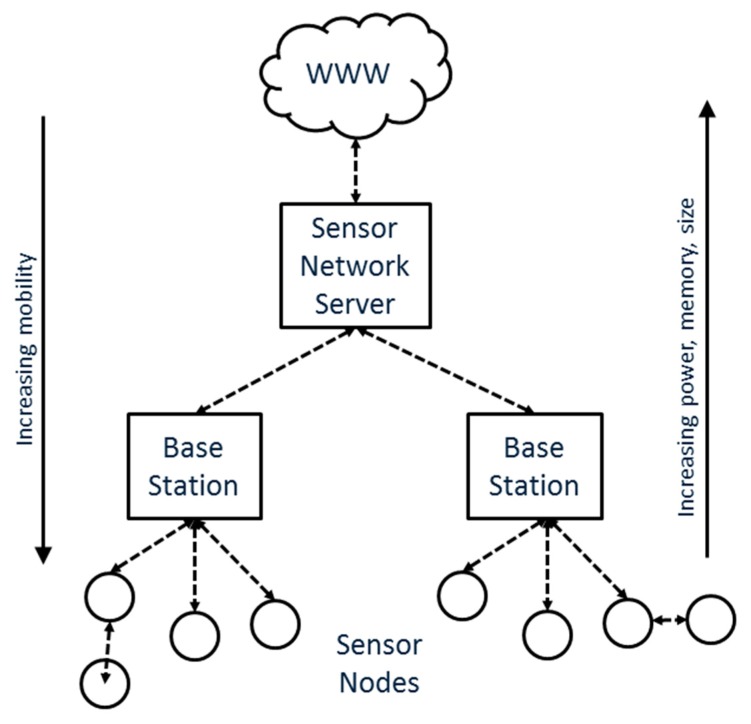
Traditional architecture of an environmental sensing network, after [4].

A conventional sensor network comprises distributed sensor nodes and sinks, where data is integrated, verified and further processed [4,43]. A server layer (sensor network server) on top of the sinks acts as a gateway for distribution of the data over the Internet (Figure 2). Interoperability in this setup is achieved at the level of the sensor network server which acts as a gateway to all sensor nodes that belong to its infrastructure. Access to the observations of the sensor nodes is only achieved through the centralized gateway which determines the access conditions for the sensors and their observations. By arranging the network like that the service and sensing components are kept separate connected through an Extract-Transform-Load (ETL) component storing observations in a database which is then used for serving the data. The ETL component requires prior knowledge of the hardware which is being used and is customised individually for each sensor network.

Emerging trends in the creation and mass use of cheap and low energy consuming mobile devices described in Section 1 have the potential to considerably change this typical architecture by providing service functionality directly on each node. This allows the creation of a distributed network of sensors on an “atomic” level materializing the vision that sophisticated services can then be created at any layer of the sensor network infrastructure, even at the device level [45]. As a result any node might become an independent element within SOAs. We expect that this reuse of the SOA concept on a node/gateway level will provide significant advantages over the traditional architecture by improving the peer-to-peer communication with the device and the flexibility of data discovery and delivery. In addition, the use of well-established standards and technology act together as enablers of the IoT.

**Figure 3.**
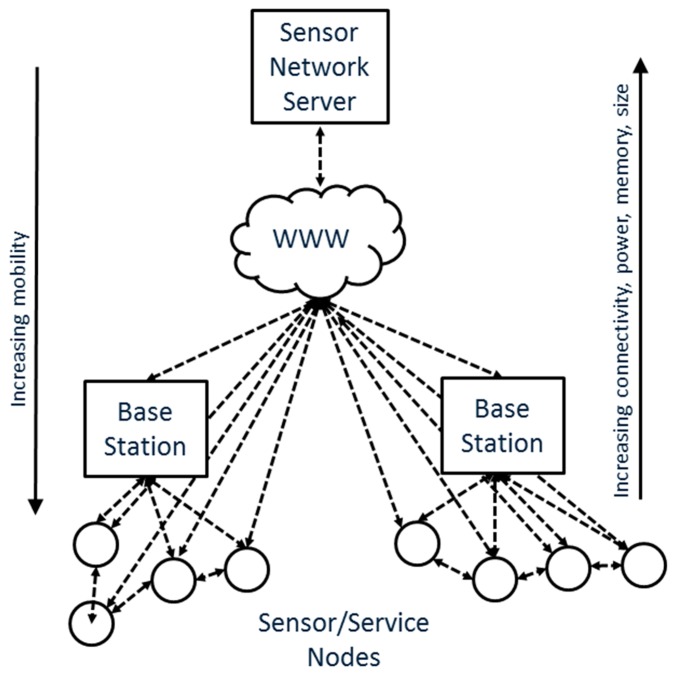
Distributed SOA architecture with on-board discovery, serving and sensing functionality.

A “service-enabled” sensor can through this architectural setup easily be allocated to more than one sensor network (Figure 3). In a traditional environmental sensing network such flexibility would be difficult to achieve, as at least prior knowledge of the sensor and the ETL procedure would be needed, if data integration to more than one sensor network is desired. This does not eliminate the need of middleware where crucial services should continue to be deployed and data from one or more sensor can be verified, aggregated, restructured and served. The use of on-device services would however provide a more flexible approach and improve the access and opportunities for reusing observation data from sensors. This has the potential to hide the heterogeneity of devices and protocols behind a web service layer on the lowest possible level of the sensor nodes.

**Figure 4.**
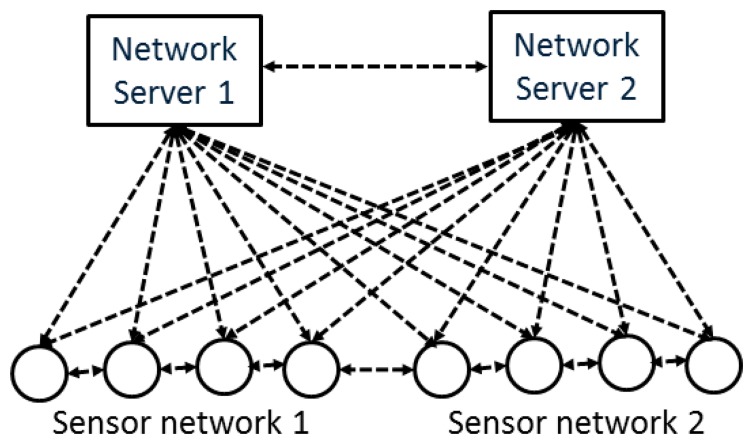
Interaction between two sensor networks.

Embedding services in individual sensor nodes also allows setting up networks in a flexible way, allowing a node to be flexibly assigned to more than one network without preliminary knowledge of the hardware or specific protocols being used (Figure 4).

### 3.2. Applicable Standards

We see the ability to provide web services on each sensor node as a precondition for the reuse of observation data and the establishment of flexible sensor networks. However, the interfaces and data formats of the services running on these nodes can be different from each other. This may lead to difficulties combining the web services between different networks or integrating them into different application tools. The use of commonly agreed standards creates opportunities to solve this change and to facilitate the reuse of data coming from different sensors. The following standards, already introduced in Section 2.4.3, if implemented on a sensor node considering the computational capabilities and objectives of the node, can significantly improve interoperability of the sensor network and between sensor networks:

- *Data encoding (O&M and JSON).* Data encoded as O&M can directly be used in a geographic information system (GIS) or be served through an SOS server. Currently there is ongoing work to create JSON encodings [46] in addition to the already available XML encodings for the O&M data model.
- *Sensor metadata (SensorML).* SensorML allows the provision of metadata about sensors and measurement processes in a common XML format. Also in this case a JSON representation is currently in preparation.
- *Data serving (SOS)*. Data encoded as O&M and/or in a lightweight JSON format can be accessed through the SOS interface. Currently there is ongoing work to also define a RESTful SOS interface in addition to the existing HTTP GET and POST bindings.
- *Triggering a notification (SES).* Specific functionalities (e.g., sending notifications) can be triggered through an SES if a user-defined event occurs (e.g., a threshold value is reached). This is advanced by the currently ongoing OGC Publish/Subscribe working group which aims to advance ideas from the SES discussion paper to a stable OGC standard.

In addition, when applying the O&M, SensorML and the SOS standards, it is important to note that these standards offer a quite comprehensive set of optional elements and operations. To increase interoperability it is necessary to ensure that an agreed minimum set of elements and operations is supported by SOS servers. For this purpose, in the OGC community a lightweight profile for the SOS interface was developed [47]. After further advancement and optimisation this profile was included in a Best Practice Paper [48]. Within our proposed approach, we recommend this profile of the SOS server to ensure the highest possible level of interoperability.

### 3.3. Proof of Concept

We built a prototype environmental sensing platform based on low-cost hardware, bundled with FOSS software. Following the deployment and configuration of the necessary components we executed several productivity tests in order to ensure that the device performs in accordance with the expectations. This section of the manuscript provides an overview of the components which we used, as well as synthesised results from our experiment.

#### 3.3.1. Hardware

##### Characteristics

Following a review of the available single-board computers we chose the Raspberry PI (model B) device [23] for our proof-of-concept. We chose this particular minicomputer because of its low energy consumption, combined with high computational power. Moreover it provided a generic Linux-based architecture through the preloaded Raspbian Linux operating system [49]. The main characteristics of the device are illustrated in Table 2.

When compared to other similar devices (*cf.* Section 2.2), the RaspberryPI consumes more energy, because of its higher computational capabilities. It is possible to power the device from a 5 V battery pack or through solar panels. In both cases the energy consumption would strongly vary depending on the usage, most of all in terms of the connected sensors, other periphery and the network utilization. Table 3 details the energy consumption model of the RasperryPI model B unit, under several different operational modes.

##### Costs

The RaspberryPI, as a non-profit project which is being created by a UK-based foundation [23] comes at a price which is considerable lower than the rest of the similar projects described in Section 2.2 (Table 4). The device itself costs only EUR 25, and the overall node cost would depend on the additional components such as a sensor shield, case, *etc.* In terms of sensing capabilities the device can be connected to a wide variety of sensors either through pre-packaged bundles, e.g., the AirPi for monitoring the atmospheric conditions (incl. temperature, relative humidity, air pressure, UV levels, illumination, nitrogen dioxide, carbon monoxide, and smoke level), or through individual components. With the latter option the price can be kept considerably lower, whereas the former provides an easy to use plug-and-play means for establishing a sensor node. Additionally, a connection bridge is available which allows the device to be connected to an Arduino (and possibly Waspmote, which would require additional testing).

#### 3.3.2. Software

Following the choice of appropriate hardware we tested the deployment of generic open source components, considering that the RaspberryPI has originally been created for completely different purposes. We deployed software components in order to establish an interoperable service-enables sensor node, comprising of the following:

- Database: PostgreSQL (incl. the PostGIS spatial extension);
- Servlet Container: Apache Tomcat 6;
- Sensor Observation Service: (52°North SOS 4.0).

The advantage of this solution over other possible implementations using Arduino or Waspmote is that all those software components can be used “out-of-the box”, *i.e.*, without the need of additional development work or use of a particular device-specific IDE. Raspberry Pi Foundation [23] as vendor of the device, recommends Python as the preferred programming language. Any other language which compiles for ARMv6 can be used with the Raspberry Pi. Furthermore there are numerous industry standard programming languages such as C, Ruby, C++ and Java which are provided by default within the Raspbian Linux operating system.

#### 3.3.3. Performance

**Figure 5.**
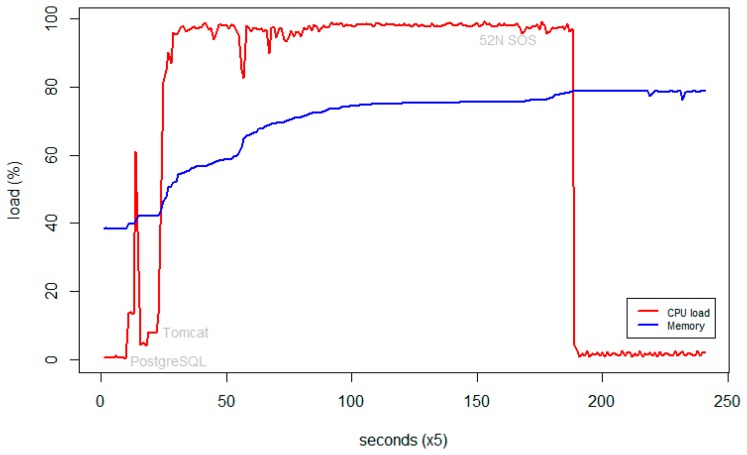
RaspberryPI memory and processor loads prior to optimization.

We tested the performance of the interoperable sensor node in order to ensure that the device can work properly when loaded with the necessary software, described in Section 3.3.2. We executed a series of productivity tests for monitoring the load of the central processing unit (CPU) and memory. Figure 5 provides information about the performance of the device after consequently starting PostgreSQL, Tomcat and the SOS server.

With the default Raspbian settings the memory utilization was high, stabilizing around 79% after running the necessary daemons (Figure 5). We considered that this might cause problems with the productivity of the device under operational conditions, and particularly in cases when the sensor node should both collect data and serve multiple requests. That is why we attempted to optimize the default operating system parameters. The following steps were consequently accomplished in order to reduce the memory usage and improve the performance of the processor:

- Disabled graphical user interface (GUI);
- Enabled memory swap space (512 MB);
- Processor overclocking (from the default 700 MHz to 850 MHz).

**Figure 6.**
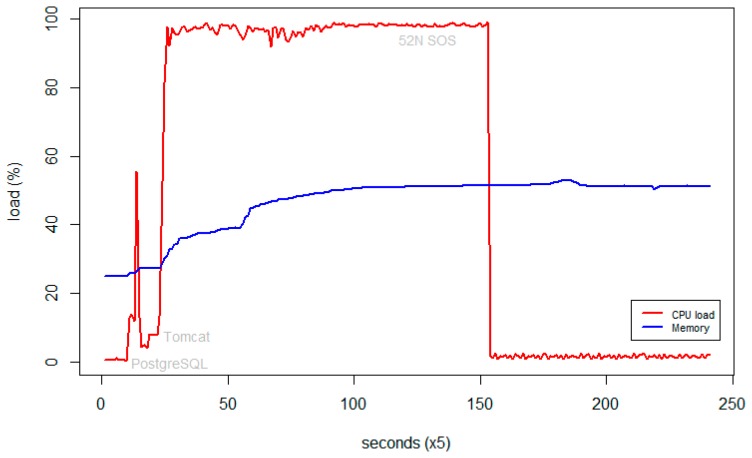
RaspberryPI memory and processor loads after optimization.

This did lead in an overall reduction of the memory usage by approximately 35%, combined with a shortened time for computation due to the overclocked processor (Figure 6).

As result of the experiment, and the tests which we executed we consider that the combination of software and hardware as described above can be used in order to establish a service-enabled sensor node, thus making the proposed architecture possible. Moreover, we use this prototype only as an example which illustrates that the availability of highly productive and affordable mobile hardware, might change the traditional ways of environmental sensing through the following:

- Massive deployment of cheap devices (also by citizens), which are not likely to substitute the existing authoritative data sources, but create complementary (or alternative) data streams;
- High computational capabilities kept at sensor node, which was not possible until recently;
- Interoperable means of data provision, allowing multiple accesses to data without preliminary knowledge of the actual technology, *i.e.*, the manufacture-specific details and characteristics can be “hidden” behind an interoperability “layer” which is based on well-established international standards (OGC, ISO, ITU, *etc.*).

## 4. Use Case Scenarios

In order to illustrate the value and innovation potential of the proposed interoperable architecture, we provide several examples from different domains, where we envision significant gains stemming from the deployment of direct sensor node access protocols, through standardized http requests.

### 4.1. Precision Farming

Environmental sensing for monitoring of agro-ecological conditions, for phenomena such as soil moisture, temperature and humidity, is increasingly important, as it provides the means for sustainable farmland management. In this context, a cost efficient plug-and-play sensor device capable of broadcasting data in an interoperable manner would allow farming communities to better observe the agro-ecological conditions. Furthermore, once collected and processed, such information can be reused in completely different contexts, such as atmospheric modelling or environmental protection.

### 4.2. Atmospheric Monitoring

Besides the obvious advantage that an interoperable solution can bring along for the dissemination of meteorological data, it can be used for a broad spectrum of applications dealing with monitoring the state of the atmosphere, such as environmental noise, parameters of air quality and odour. For instance, in a typical urban scenario, local authorities could deploy inexpensive in-situ sensor nodes for monitoring the industrial facilities which emit atmospheric pollutants. Similar approaches can be envisioned for sensor-based odour monitoring. Numerous applications can then be developed on top of the interoperable services, including:

- Web applications for citizens living in proximity of industrial sites, so as to evaluate the air quality in their neighbourhood;
- Harvesting applications for the preservation of historic data for scientific purposes (e.g., interpolation or correlation with meteorological data, *etc.*);
- Applications for environmental protection agencies and local authorities which trigger an alarm if one or more of the sensor nodes report exceedance of a pollutant above a predefined threshold.

### 4.3. Smart Cities

The rapid increase of the relative share of world's population living in cities, together with the demand for smarter and more sustainable decisions related to the urban environment require larger than ever volumes of heterogeneous data to be quickly collected, processed, analysed and communicated. Moreover, information is during the past 20 years among the major factors enhancing the competitive profile of a city [50]. Given this context, the proposed architecture would lead to an improvement of the access to observations. They can be acquired directly from the devices that produced the data. The observations can then be reused in numerous different contexts. Possible applications include observation of environmental conditions in urban areas, asset management, traffic related data collection, and many others.

### 4.4. Indoor Conditions Monitoring

Devices which are kept indoors can relatively easily be connected to network and power supply, and then provide a means for monitoring the conditions for different parameters such as illumination, humidity, and temperature. As the nodes in the proposed architecture are service-enabled a value-added solution can be developed on top of the infrastructure, for instance, to trigger a process once a threshold value is reached. An example application in that sense can be a gallery or a museum where interoperable devices are connected to the IoT and send an email, SMS or tweet when humidity reaches a predefined value (of e.g., 70%), thus threatening artwork and requiring intervention from maintenance personnel. Moreover, as the service sending data is interoperable such value-added solutions can easily be reused in completely different conditions, for instance in hospitals, schools, theatres or museums to ensure that the indoor conditions are optimal.

## 5. Discussion and Conclusions

In this paper, we have presented a holistic vision of the main challenges of mobile sensing, explore existing standards and technologies in the context of the IoT, and introduce a platform which enables interoperable sensing applications on the Web. We have reviewed a large amount of research work and sensing technologies, analysed technical issues and key aspects in the design of interoperable sensing networks. We have described how recent advancements in the fields of standardization and hardware could play an important role in improving the access to environmental information from sensors in the context of the IoT. In the first section we have provided a synthesized view of the factors which—in their combination—can lead to better data access. In the second section, we have identified low-cost mobile computing hardware, lightweight open source software, and well established standards as the empowering factors for interoperable environmental sensing. The service-enabled sensor node, described in the third section would have implications to cities and their citizens in an increasingly dynamic world, as they would provide a “plug-and-play” solution, which is ready to observe and serve data that can easily be combined and reused for decision making, in near real-time. However, issues such as energy consumption and Internet connectivity still represent critical technical barriers for collecting and processing sensor data. Current mobile phones and wireless sensor technologies (or single add-on sensors or embedded sensors in mobile phones) must overcome the existing limitations of *Bluetooth*/*Wi-Fi* connectivity to form efficient multi-hop communication networks. Research trends on device-to-device communications, or context-aware algorithms could improve the current *ad hoc* communication among devices, as well as the overall energy efficiency.

To conclude, the ability of sensor nodes to also serve observation data, as described in this manuscript raises the following questions, which would determine our future research direction:

- Scalability and productivity

Even though sensor nodes increasingly become computationally capable, the considerable portion of devices is still not powerful enough to perform complex operations. The SensorThingsAPI candidate OGC standard addresses exactly this matter by providing an open and unified framework to interconnect IoT devices, data, and applications over the Web [51]. The SensorThingsAPI has the potential to provide a single access paradigm to millions of sensing devices (things), which can be intuitively used by developers in order to create value-added products. This candidate standard particularly addresses ease of use within and outside the SWE and SDI community. It provides a REST-like interface for querying things and their observations, together with a JSON encoding of a subset of the O&M data model, which has been simplified and extended by an IoT-fit model. It might equally serve raw measurements as well as derived information, such as spatial or temporal aggregates. Possible architectural set-ups and user-specific applications remain to be investigated while the standard still has to be formally submitted to the OGC adoption process. The authors of this paper are considering contributing their experiences gained from the analysis described in this article to the advancement of the SensorThingsAPI to make it suitable for further application scenarios. Compared to the conventional OGC SWE standards, the SensorThingsAPI offers a much more lightweight approach but also misses some elements (e.g., an approach such as the SOS Spatial Filtering Profile to cover the specific needs of mobile sensor networks). At the same time it should be investigated how the use of such a standardised approach can facilitate the integration with other IoT platforms (e.g., OpenIoT).

- Data aggregation

On-device interoperability, once achieved, would enable flexible data aggregation by following unprecedented new patterns. For instance, aggregation frameworks could be provided on top of the unified interfaces and data models and would thereby become portable across vendor-specific solutions. The ability to deploy processing capabilities close to the sensors allows aggregations to be computed next to the original (raw) data and thus reduce data traffic in the following value chains. These aggregations might be offered as Observations and Measurements documents via an SOS server or the SensorThingsAPI again, which would lead to a nested sensor network. Calibrations would even allow to make these sensing devices “programmable” and thus customisable for particular application areas. Proof of concepts are however yet to be developed.

- Semantic interoperability

This article presents the overall approach and thus advances technical interoperability by introducing standardized web service interfaces as close as possible to sensing devices. Heterogeneities of transfer protocols, formats and data modelling are addressed on a syntactical and structural level. Semantic interoperability is only partially addressed by well-established existing code lists and highly specialized datatypes that are defined as part of the SWE framework. It is well-known that this solution has its limits [52]. Especially in the IoT context, we can expect semantic interoperability challenges due to the high degree of heterogeneity in phenomena of interest, observed properties and applied measurement procedures. Possible solutions, such as the Semantic Sensor Network (SSN) Ontology of the World Wide Web Consortium (W3C) [53] have been developed during the past decade, as part of the Semantic Web and Linked Data movement. Previous work on a Linked Data Proxy for the SOS [54] enables the direct connection of the technologies that have been suggested in this article with the Semantic Web. Further investigations of the application of semantic interoperability solution have to be carried out within practical case studies.

- Implications on data analysis

Taking these considerations one step further, the overall approach towards interoperable and customisable sensor services for the IoT might heavily impact approaches for data analysis. As soon as processing becomes part of the sensor network, the pre-processing that is required to fit observations into applications, such as predictions of environmental conditions (weather, air quality, noise, *etc.*), can be directly applied when a new measurement is taken. In this way, the proposed approach removes some of the time-loss in sensor processing and supports near-real time applications. On the contrary, as already argued above, an evaluation could be implemented as part of the network, so that targeted calculations are only performed (close to the sensor) when required. This functionality might, for example, be applied to disaster situations, where analyses are only required once a potentially hazardous event takes place. Considering the area of visual analytics [55], which becomes increasingly prominent with the raise of Big Data [56], the presented solution provides a responsive sensing environment, which can serve close to real-time and computationally optimized analysis capabilities while data is exploited.

- Data quality

The increased availability of data due to the rapidly emerging IoT will not be implicitly accompanied by an equivalent data quality improvement. Moreover, the decreasing prices of devices will possibly lead to “rounder” multi-purpose sensing hardware designs, which compromise the quality of the observations in favour of versatility. In the context of the proposed architecture the establishment of a reputation framework for sensor networks, as defined by [57] is needed more than ever. Data calibration between devices and/or in near-real-time conditions should be tested with heterogeneous devices, in order to ensure minimum quality of the data output. In addition, it should be recognised that the required quality of measurements depends on the intended purpose(s). The highest accuracy of available expert sensors systems might not be required for addressing a particular scientific question. Furthermore, as for example the in the area of Citizen Science and Do-It-Yourself/ Do-It-Together sensing [58], the derivation of new scientific findings is only one motivation of using the IoT, and only here data quality is essential. Other motivations might include the evaluation of policy impacts, awareness raising and education, or entertainment.

- Privacy and security

Obviously, privacy and security concerns should be addressed together, or within standards. Measuring temperature, energy consumption or even only the lighting situation of a property already allows identification of human presence. Especially combinations of sensors and other sources can counter play most anonymization efforts. However, here, major issues have to be addressed on a cultural, legal and organisational level before offering the supporting technical solutions. On-sensor or close-to-sensor aggregation may play its role, together with device to device combination. Still, it should be mentioned that such solutions will only fall into place if they are supported by policies and enable valuable business models. While, for example, peer-to-peer communication could be easily used to inform vehicles affected by an accident on their way ahead, enterprises are much more interested on centralised solutions in which they get control of all the collected data. Maybe we could counter play such developments if IoT and sensor technology gets smart and cheap enough.

## Figures and Tables

**Table 1.** Tested software.

**Software Product**	**Description**	**Advantages**	**Disadvantages**
**1. Operating Systems**			
Raspbian	Linux distribution based on Debian, optimized for the RaspberryPI hardware	Big user community and documentation base Out-of-the box solution Full Linux-based control over the OS Built-in graphical user Interface Capabilities similar to a desktop computer	Requires optimization for resource preservation Usable only on RaspberryPI computers
TinyOS	Operating system designed for low-power wireless devices	Lightweight OS Low power consumption	Very different from mainstream OS, requiring additional preparation Support for hardware platforms is limited
Contiki	Operating system designed for low-power wireless devices	Lightweight OS Low power consumption	Support for hardware platforms is limited
**2. IoT Integrated Development Environments (IDE)**			
Arduino IDE	Environment for writing code, which is then uploaded to the Arduino I/O board.	Created especially for mobile devices	Poor error reporting capabilities
Waspmote IDE	Environment for writing code, which is then uploaded to the Waspmote board.	Based on Arduino IDE Created especially for the IoT	Limited documentation Works only with Waspmote hardware Poor error reporting capabilities (inherited from Arduino IDE)
**3. Data Storage**			
PostgreSQL/PostGIS	Spatially enabled relational database for storing observations and measurements	Big user community Rich geospatial functionality Ability to consume data directly from a Geographic Information System (GIS) Indexing	Requires Linux/Windows operating system Resource consuming
SQLite/SpatiaLite	Lightweight relational database for storing observations and measurements	Lightweight spatial database Ability to consume data directly from a Geographic Information System (GIS) File-based storage	Not supported by studied SOS implementations Poorer geospatial functionality (when compared with PostgreSQL/PostGIS)
H2	Lightweight relational database for storing observations and measurements	Lightweight spatial database File-based storage Geospatial extension available Supported by 52°North SOS 4.x	Larger memory footprint compared to SQLite (1 MB *vs.* 350 KB)
**4. Data Serving**			
52°North Sensor Observation Service (SOS)	Open source implementation of the OGC SOS interface standard	Big user community Many deployments Administration and configuration GUI Additional lightweight RESTful interface	Needs deployment through servlet container such as Apache Tomcat 6
Mapserver Sensor Observation Service (SOS)	Open source implementation of the OGC SOS interface standard	Lightweight solution Single configuration file (*.map) Runs on Apache	Limited user community Created with static geospatial data in mind

**Table 2.** Characteristics of RaspberryPI, model “B”.

**Parameter**	**Characteristics**
Dimensions	85.60 mm × 56 mm × 21 mm
Weight	45 g. (excl. case)
System on a Chip	Broadcom BCM2835
Processor	ARM1176JZFS (700 MHz, option for overclocking up to 1000 MHz)
Video core	4 GPU
RAM	512 MB
Storage	4 GB SD card (max. 32 GB)
Interfaces	USB (2×), SD card, HDMI, 3.5 mm audio jack, GPIO (26 dedicated pins)
Network	10/100 Mbps wired Ethernet Wi-Fi USB Dongle
Power supply	5 v micro USB power supply
Operating temperature range	−25 to +80 °C

**Table 3.** Energy consumption model of RaspberryPI, model “B”.

**Running Mode**	**Volt**	**Ampere**	**Watt**
Standby	5.24	0.05	0.262
Operational (GUI running)	4.97	0.38	1.8886
Operational (Wi-Fi Dongle powered)	4.97	0.43	2.1371
USB Disabled	5.08	0.15	0.762

**Table 4.** Costs and characteristics of RaspberryPI components.

**Item**	**Description**	**Price (EUR)**
**Computer**		

RaspberryPI Model “B”	Single-board credit card sized computer	25.0
SD Card	Standard Secure Digital Card with the following characteristics: Memory size: 4 GB Operating system (incl. preloaded Raspbian Linux)	10.5

**Additional Components (Optional)**		

Wi-Fi Dongle (optional)	USB IEEE 802.11 b, g, n wireless dongle	17.0
PiFace I/O (optional)	Expansion board for Raspberry PI: Relays (4×) Switchs (4×) Digital Inputs (8×) Outputs (8×) LEDs (8×)	25.4

Case (optional)	Plastic protective case	6.00

**Sensors**		

AirPi kit	Raspberry Pi weather station shield (v 1.4)	77.4
Soil moisture components	Individual components necessary for observation of soil moisture	13.7
Arduino shield connection bridge (optional)	Connection bridge, allowing to use shields, boards and modules designed for Arduino with the computational power of Raspberry Pi	40.0

## References

[1] Swan M. (2012). Sensor Mania! The Internet of Things, Wearable Computing, Objective Metrics, and the Quantified Self 2.0. J. Sens. Actuator Netw..

[2] INFSO D.4 Networked Enterprise & RFID INFSO G.2 Micro & Nanosystems (2008). Co-operation with the Working Group RFID of the ETP EPOSS, Internet of Things in 2020, Roadmap for the Future. ftp.cordis.europa.eu/pub/fp7/ict/docs/enet/internet-of-things-in-2020-ec-eposs-workshop-report-2008-v3_en.pdf.

[3] Martinez K., Hart J., Ong R. (2004). Environmental sensor networks. IEEE Comput..

[4] Hart J., Martinez K. (2006). Environmental Sensor Networks: A revolution in the earth system science?. Earth-Sci. Rev..

[5] Guinard D., Trifa V., Karnouskos S., Spiess P., Savio D. (2010). Interacting with the soa-based internet of things: Discovery, query, selection, and on-demand provisioning of web services. IEEE Trans. Serv. Comput..

[6] Atzori L., Iera A., Morabito G. (2010). The internet of things: A survey. Comput. Netw..

[7] Jirka S. (2010). Challenges of Sensor Discovery. Movement-Aware Applications for Sustainable Mobility: Technologies and Approaches.

[8] Lange S., Nettstater A., Haller S., Carrez F., Bassi A. Introduction to the Architectural Reference Model for the Internet of Things, 2012. http://www.iot-a.eu/arm.

[9] Lanley D. (2001). 3D Data Management: Controlling Data Volume, Velocity and Variety. http://blogs.gartner.com/doug-laney/files/2012/01/ad949-3D-Data-Management-Controlling-Data-Volume-Velocity-and-Variety.pdf.

[10] Havlik D., Schade S., Sabeur Z.A., Mazzetti P., Watson K., Berre A.J., Mon J.L. (2011). From sensor to observation web with environmental enablers in the future internet. Sensors.

[11] Heflin J., Hendler J. (2000). Semantic Interoperability on the Web.

[12] Motwani R., Motwani M., Harris F., Dascalu S. Towards a Scalable and Interoperable Global Environmental Sensor Network Using Service Oriented Architecture.

[13] Gutierrez J.A., Winkel L., Callaway E.H., Barrett R.L. (2011). Low-Rate Wireless Personal Area Networks: Enabling Wireless Sensors with IEEE 802.15.4.

[14] IEEE Computer Society, IEEE Standard IEEE 802.15.4–2011 v8.1.2.2 2011. http://standards.ieee.org/getieee802/download/802.15.4-2011.pdf.

[15] Gracanin D., Eltoweissy M., Wadaa A. (2005). DaSilva. L.A. A service-centric model for wireless sensor networks. IEEE J. Sel. Areas Commun..

[16] International Telecommunication Union (ITU) Internet of Things Recommendation ITU-T Y.2060 (06/2012). https://www.itu.int/rec/dologin_pub.asp?lang=e&id=T-RECY.2060-201206-I!!PDF-E&type=items.

[17] International Telecommunication Union (ITU) Joint Coordination Activity on Internet of Things (JCA-IoT), Deliverable 1, Generic Reference Model Architecture. http://www.itu.int/en/ITU-T/jca/iot/Pages/default.aspx.

[18] Digital Agenda—Network Technologies. http://ec.europa.eu/digitalagenda/en/networktechnologies.

[19] Digital Agenda—Towards 5G Networks. http://ec.europa.eu/digital-agenda/en/towards-5g.

[20] Google Loon Project Overview. http://www.google.com/loon.

[21] Overview of the Internet.org Initiative. http://internet.org.

[22] Priyantha B., Kansal A., Goraczko M., Zhao F. Tiny Web Services for Sensor Device Interoperability.

[23] RaspberryPI foundation. www.raspberrypi.org.

[24] Upton E., Halfacree G. (2012). Meet the Raspberry Pi.

[25] Reusing T. (2012). Comparison of operating systems TinyOS and Contiki. Sens. Nodes-Oper. Netw. Appl. (SN).

[26] Arduino Platform Homepage. www.arduino.cc.

[27] Waspmote Sensor Platform. www.libelium.com/products/waspmote_.

[28] Scacchi W., Feller J., Fitzgerald B., Hissam S., Lakhani K. (2006). Understanding free/open source software development processes. Softw. Process Improv. Pract..

[29] Erl T. (2005). Service-Oriented architecture: Concepts, technology, and design. Pearson Education India.

[30] Höller J.V., Tsiatsis C., Mulligan S., Karnouskos S., Avesand D. (2014). Boyle: From Machine-to-Machine to the Internet of Things: Introduction to a New Age of Intelligence.

[31] Technical Specifications of Bluetooth Low Energy. www.bluetooth.com/Pages/low-energy-tech-info.aspx.

[32] Thread Wireless Protocol—Homepage. http://news.silabs.com/press-release/introducing-thread-new-wireless-networking-protocol-home.

[33] Overview of Bluetooth Smart. www.bluetooth.com/Pages/Bluetooth-Smart.aspx.

[34] Z-Wave Alliance Homepage. www.z-wavealliance.org.

[35] Z-Wave Homepage. www.z-wave.com_.

[36] OpenIoT Project Homepage. www.openiot.eu_.

[37] Van der Schaaf H., Herzog R. OGC SensorThings API Implementation on the OpenIoT Middleware. SoftCOM 2014 workshop on Interoperability and Open-Source Solutions for the Internet of Things, Split, 2014. http://marjan.fesb.hr/SoftCOM/2014/.

[38] Dimitropoulos P. (2012). OpenIoT Platform Requirements and Technical Specifications. http://openiot.eu/sites/all/themes/corporateclean/Files/OpenIoT_D22.pdf.

[39] Directive 2007/2/EC of the European Parliament and of the Council of 14 March 2007 establishing an Infrastructure for Spatial Information in the European Community (INSPIRE). http://eur-lex.europa.eu/legal-content/EN/TXT/?qid=1400687098123&uri=CELEX:32007L0002.

[40] INSPIRE (2013). D2.9 Guidelines for the use of Observations & Measurements and Sensor Web Enablement-related standards in INSPIRE Annex II and III data specification development. http://inspire.jrc.ec.europa.eu/documents/Data_Specifications/D2.9_O&M_Guidelines_v2.0rc3.pdf.

[41] Bröring A., Jirka S., Kotsev A., Spinsanti L. Making the Sensor Observation Service INSPIRE Compliant, INSPIRE Conference, 2013. http://inspire.jrc.ec.europa.eu/events/conferences/inspire_2013/schedule/submissions/236.pdf.

[42] Botts M., Percivall G., Reed C., Davidson J. (2008). OGC^®^ Sensor Web Enablement: Overview and high level architecture. GeoSens. Netw..

[43] Akyildiz I.F., Su W., Sankarasubramaniam Y., Cayirci E. (2002). Wireless sensor networks: A survey. Comput. Netw..

[44] Bröring A., Echterhoff J., Jirka S., Simonis I., Everding T., Stasch C., Liang S., Lemmens R. (2011). New Generation Sensor Web Enablement. Sensors.

[45] Spiess P., Karnouskos S., Guinard D., Savio D., Baecker O., Souza L., Trifa V. (2009). SOA-Based Integration of the Internet of Things in Enterprise Services. Web Serv..

[46] Overview of the JavaScript Object Notation. www.json.org.

[47] Jirka S., Stasch C., Bröring A. (2011). OGC Discussion Paper: Lightweight SOS Profile for Stationary In-Situ Sensors (OGC 11–169).

[48] (2014). EO2HEAVEN Consortium. OGC Best Practice: Best Practice for Sensor Web Enablement: Provision of Observations through an OGC Sensor Observation Service (SOS) (13–015).

[49] Raspbian Linux Distribution Homepage. www.raspbian.org_.

[50] Caragliu A., del Bo C., Nijkamp P. (2011). Smart cities in Europe. J. Urban Technol..

[51] SensorThings API OGC candidate standard. http://ogc-iot.github.io/ogc-iot-api/faq.html.

[52] Schade S., Brox C., Krüger A., Simonis I. Sensors on the Way to Semantic Interoperability.

[53] Compton M., Barnaghi P., Bermudez L., García-Castro R., Corcho O., Cox S., Graybeal J., Hauswirth M., Henson C., Herzog A. (2012). The SSN ontology of the W3C semantic sensor network incubator group. Web Semant. Sci. Serv. Agents World Wide Web.

[54] Janowicz K., Broering A., Stasch C., Schade S., Everding T., Llaves A. (2011). A RESTful Proxy and Data Model for Linked Sensor Data. Int. J. Dig. Earth.

[55] Keim D.A., Kohlhammer J., Ellis G., Mansmann F. Mastering the Information Age-Solving Problems with Visual Analytics; Florian Mansmann: 2010. http://www.vismaster.eu/wp-content/uploads/2010/11/VisMaster-book-lowres.pdf.

[56] Douglas J., Usländer T., Schimak G., Esteban J.F., Denzer R. (2008). An open distributed architecture for sensor networks for risk management. Sensors.

[57] Ganeriwal S., Balzano L.K., Srivastava M.B. (2008). Reputation-based framework for high integrity sensor networks. ACM Trans. Sens. Netw..

[58] Haklay M., Sui D., Elwood S., Goodchild M. (2012). Citizen Science and Volunteered Geographic Information—Overview and Typology of Participation.

